# Quantitative Profiling of Brain Lipid Raft Proteome in a Mouse Model of Fragile X Syndrome

**DOI:** 10.1371/journal.pone.0121464

**Published:** 2015-04-07

**Authors:** Magdalena Kalinowska, Catherine Castillo, Anna Francesconi

**Affiliations:** Dominick P. Purpura Department of Neuroscience, Albert Einstein College of Medicine, Bronx, New York, New York, United States of America; University of Minnesota, UNITED STATES

## Abstract

Fragile X Syndrome, a leading cause of inherited intellectual disability and autism, arises from transcriptional silencing of the FMR1 gene encoding an RNA-binding protein, Fragile X Mental Retardation Protein (FMRP). FMRP can regulate the expression of approximately 4% of brain transcripts through its role in regulation of mRNA transport, stability and translation, thus providing a molecular rationale for its potential pleiotropic effects on neuronal and brain circuitry function. Several intracellular signaling pathways are dysregulated in the absence of FMRP suggesting that cellular deficits may be broad and could result in homeostatic changes. Lipid rafts are specialized regions of the plasma membrane, enriched in cholesterol and glycosphingolipids, involved in regulation of intracellular signaling. Among transcripts targeted by FMRP, a subset encodes proteins involved in lipid biosynthesis and homeostasis, dysregulation of which could affect the integrity and function of lipid rafts. Using a quantitative mass spectrometry-based approach we analyzed the lipid raft proteome of Fmr1 knockout mice, an animal model of Fragile X syndrome, and identified candidate proteins that are differentially represented in Fmr1 knockout mice lipid rafts. Furthermore, network analysis of these candidate proteins reveals connectivity between them and predicts functional connectivity with genes encoding components of myelin sheath, axonal processes and growth cones. Our findings provide insight to aid identification of molecular and cellular dysfunctions arising from Fmr1 silencing and for uncovering shared pathologies between Fragile X syndrome and other autism spectrum disorders.

## Introduction

Genetic studies have uncovered hundreds of candidate *loci* [1, 2] associated with Autism Spectrum Disorders (ASDs). Despite remarkable progress, one major outstanding challenge is identification of shared biological pathways that could contribute to convergent dysfunctions arising from genetic heterogeneity [3, 4]. Fragile X syndrome (FXS), a leading cause of inherited intellectual disability, accounts for 2–5% of autism cases and ~30% of individuals with FXS are diagnosed with autism, suggesting that the conditions may share common pathophysiology [5, 6]. FXS is a monogenic disorder that arises from abnormal expansion of a tri-nucleotide repeat in the 5’-untranslated region of the X-linked Fmr1 gene, leading to transcriptional silencing that results in loss of Fragile X Mental Retardation Protein (FMRP) expression [7]. FMRP is an RNA-binding protein that binds to >4% of brain transcripts [8, 9] to regulate mRNA translation, trafficking and stability [7, 10–12]. FMRP was shown to inhibit mRNA translation [7] and in its absence basal protein synthesis is abnormally elevated [13, 14]. Profound abnormalities in intracellular signaling pathways have also been observed in cells lacking FMRP. Cyclic AMP production is decreased [15, 16] whereas basal activities of Ras [17], extracellular signal-regulated protein kinases 1/2 (ERK1/2; [18, 19]), phosphatidylinositol-4,5-bisphosphate 3-kinase (PI3K; [20, 21]) and mammalian target of rapamycin (mTOR [22, 23]) are abnormally enhanced. In contrast, receptor dependent signaling is impaired including signaling by group 1 metabotropic glutamate receptors (Gp1 mGluRs) to ERK1/2 [19] and PI3K-AKT-mTOR [22, 24] pathways and signaling by dopamine receptor 1 (D1R) to the cAMP cascade [25]. Collectively, these findings suggest that loss of FMRP leads to general deficits in the efficiency and regulation of cellular signaling, dysfunctions that might be common to other disorders in the autism spectrum.

Lipid rafts (also termed membrane rafts) are liquid-ordered regions of the plasma membrane, originating from local enrichment in glycosphingolipids and cholesterol, that recruit selected integral membrane proteins and proteins carrying glycosylphosphatidylinositol (GPI) anchors: in addition, palmitoylated and acylated proteins associate with the inner leaflet of plasma membrane rafts [26, 27]. Lipid rafts are thought to provide transient ‘platforms’ for receptors and signaling effectors thereby contributing to regulation of cell signaling [28, 29]. Native lipid rafts are plastic, transient, small (~40–100 nm) membrane subdomains that are not amenable to biochemical purification; description of their putative composition as well as identification of properties shared by proteins endowed with raft affinity have largely originated from the characterization of detergent-resistant membranes (DRMs). DRMs are a collection of buoyant membranes resistant to solubilization by cold, non-ionic detergents and are selectively enriched in cholesterol, glycolipids and raft-associated proteins [30]. Importantly, DRMs are amenable to biochemical purification and thus afford a means of assessment of lipid raft composition under basal and perturbed conditions [31].

In the central nervous system (CNS), lipid rafts have been implicated in establishment of neuron polarity [32, 33], axon guidance [34], dendritogenesis [35], spine morphogenesis [36] and myelination [37, 38]. Lipid raft composition is remodeled during neuronal differentiation [34] and aging [39], and is altered in neuropsychiatric conditions [40] including Alzheimer’s disease [41], schizophrenia [42], depression [43] and Smith-Lemli-Opitz syndrome [44]—an autism spectrum disorder [45, 46]. Despite the broad impact of Fmr1 ablation on neuronal signaling, it remains unknown whether the composition of lipid rafts is altered in animal models of FXS. A subset of RNA transcripts predicted to bind FMRP [8] encodes proteins critically involved in lipid synthesis and transport: thus, loss of FMRP expression may conceivably affect lipid homeostasis and lipid raft properties.

To examine whether loss of FMRP expression results in alterations in the properties of lipid rafts, we used a systems biology approach based on protein identification and quantification by mass spectrometry. We used isobaric tags for relative and absolute quantification (iTRAQ; [47]) to label peptides for qualitative and quantitative analysis of the proteome of DRMs purified from mouse brain. By this strategy we uncovered candidate proteins that are differentially represented in the lipid raft proteome of FMR1 knockout mice and linked them by network analysis to specific cellular components and functional pathways, such as axonal processes and myelination, which could be affected by ablation or silencing of Fmr1.

## Materials and Methods

### Reagents and animals

The following antibodies were used according to manufacturer recommendations: mouse monoclonal antibodies anti-Flotilllin-1 (BD Biosciences), anti-Homer1b/c (Santa Cruz Biotechnology), anti-Ly6h (Abnova) anti-Plp1 (Millipore), anti-Thy-1 (BioLegend), anti-Transferrin receptor 1 (Invitrogen), anti-β-Tubulin (Sigma Aldrich), anti-γ-Tubulin (Sigma Aldrich) and rabbit polyclonal antibodies anti-CaMKIIα/β Cell Signaling Technology) and anti-Gnb2 (Abcam). Mouse anti-Versican core protein (clone 351/24) was obtained from the UC Davis/NIH NeuroMab Facility. Wild type and Fmr1 null (FVB.129P2-*Pde6b* ^*+*^ strain) mice were obtained from The Jackson Laboratories (Bar Harbor, ME) and bred in house. For euthanasia, mice were deeply anesthetized by isoflurane inhalation until insensate and decapitated. All animal procedures were carried out according to protocols approved by the Albert Einstein College of Medicine Institutional Animal Care and Use Committee and in accordance with the Guide for the Care and Use of Laboratory Animals by the United States Public Health Service.

### Subcellular fractionation and western blot

Cortices (hippocampi removed), hippocampi and cerebella from adult WT and Fmr1 KO mice (~ five month-old) were homogenized on ice in homogenization buffer of 10 mM Tris-HCl pH 7.4, 5 mM EDTA, 320 mM sucrose, eukaryotic protease inhibitor cocktail and Na_3_VO_4_. Homogenates were centrifuged for 10 min at 800 x *g* to precipitate nuclei and supernatant fraction collected (S1) and centrifuged at 10,000 x *g* for 15 min. The resulting pellet (P2) and supernatant (S2) were separated and solubilized in lysis buffer containing 50 mM Tris-HCl pH 7.4, 150 mM NaCl, 1 mM EDTA, 1% Triton X-100, 0.5% CHAPS supplemented with protease inhibitor cocktail and Na_3_VO_4_. Analysis of proteins was performed by protein separation by SDS-PAGE followed by immunoblot with specific primary antibodies and detection with horseradish peroxidase-conjugated secondary antibodies as previously described [48].

### Isolation of detergent resistant membranes (DRM) from mouse brain tissue

DRM preparation was carried out according to previously described procedures [49]. Briefly, adult mice (~ five to six month-old) were euthanized and microdissected tissue rinsed with saline solution and homogenized with a Dounce tissue grinder in cold homogenization buffer with protease inhibitors. After low speed centrifugation to remove nuclei and residual particulate material, supernatants were spun at 10,000 x *g* at 4ºC and resulting pellets dissolved in cold buffer of 50 mM Tris-HCl pH 7.4, 150 mM NaCl, 5 mM EDTA, 1% Triton X-100 (v/v) and incubated on ice (10 min). Solubilized pellets were adjusted to 40% sucrose and fractionated on discontinuous sucrose gradients by centrifugation at 34,000 rpm (SW40 Ti; Beckman Coulter) for 16 h. Thirteen fractions (1 ml each) were collected from each gradient starting from the top and an aliquot of each analyzed by Western blot.

### iTRAQ proteomics

We carried out proteomic profiling of WT and Fmr1 KO mice using purified DRMs labeled with iTRAQ reagents: samples were processed by Applied Biomics (Hayward, CA) for isotope labeling and analytical mass spectrometry. Briefly, DRMs were purified from freshly dissected cortices (hippocampi removed) from individual mice (three animals per genotype in one experiment and two animals per genotype in two experiments): fractions enriched in DRMs (routinely fractions # 3, 4, and 5) were combined for each genotype to yield approximately 6 or 9 ml collective volume, depending on number of animals, and concentrated. Equal amounts of protein for WT and Fmr1 KO mice, approximately 6 to 8 μg depending on the preparation, were reduced and alkylated followed by digestion with trypsin and labeling with iTRAQ reagents (AB Sciex) with reporter ions/isobaric tags 114/113 or 118/117. Tagged peptides were combined and fractionated on a nano-LC column and analyzed by MS and MS/MS with ABSCIEX 5800 (AB Sciex). For protein identification, MS/MS spectra were submitted for search of the SwissProt and decoy databases using MASCOT. For each protein hit, only peptides with a confidence interval (C.I.) >95% were considered. Peptides corresponding to keratin, a potential contaminant, were excluded from final combined analysis of independent experiments. The ratios of peptide quantities expressed as signal intensity ratios of reporter groups for individual peaks were obtained to calculate average ratio(s) for the protein, number of peptides that contributed to the average and the geometric standard deviation. In some instances, a ratio could not be determined because one or more relevant peaks were missing or peptide match was rejected based on confidence interval.

### Gene ontology enrichment and protein interaction network analyses

Gene ontology (GO) analysis was carried out with the Database for Annotation, Visualization and Integrated Discovery (DAVID; [50, 51]). The database was searched using a subset of the brain raft proteome that included 85 genes encoding proteins for which comparative quantitative information was obtained, and used to assess enrichment in particular GO terms. Identified GO terms were grouped based on functional annotation for biological process (GO_BP), cellular component (GO_CC), and molecular function (GO_MF). Functional annotation was carried out according to published protocols [50] with default settings and using *Mus musculus* genome as background reference database. Terms with Benjamini Hochberg-corrected *p* value <0.05 are illustrated. Network analysis was carried out using a query list of four genes encoding proteins that showed differential abundance in the brain raft proteome of Fmr1 KO mice using *Mus musculus* as reference. The query list did not include Tubb2a and Tubb5, encoding tubulin isoforms, to prevent skewing the network and Ly6h due to paucity of information for this gene. Analysis and representation of network connections were carried out with GeneMANIA [52] with default settings: predicted connectivity is illustrated by links which reflect degree of confidence of relationships between gene pairs.

## Results

### Characterization of mouse brain lipid raft proteome

**Fig 1:**
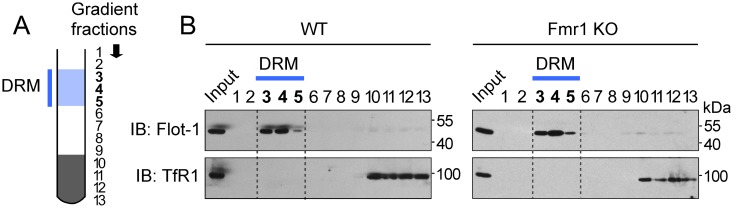
Purification of lipid raft enriched membranes from wild type and Fmr1 mutant mouse brain. A) Schematic of isopycnic sucrose gradient used in isolation procedure of detergent resistant membranes (DRMs). Light gradient fractions containing DRMs (fractions 3 to 5) and heavy gradient fractions containing non-raft proteins are indicated. B) Representative immunoblots of extracts from WT and Fmr1 KO mouse brain illustrating isolation of DRMs used for proteomic studies. Numbers above gel lanes indicate gradient fractions (#1, first fraction; #13, bottom gradient fraction). Input, total homogenate; Flot-1, Flotillin-1; TfR1, Transferrin receptor 1; IB, immunoblot.

In neurons, lipid rafts recruit ion channels and ionotropic neurotransmitter receptors, including glutamate and nicotinic acetylcholine receptors, and regulate the activity of metabotropic receptors for neurotransmitters and neuromodulators [53]. In Fmr1 null neurons, tonic activity of selected signaling effector proteins and metabotropic functions of Gp1 mGluRs, dopamine and cannabinoid [54] receptors among others are dysregulated, but whether the integrity and/or function of lipid rafts are compromised in absence of FMRP is unknown. Here, we set out to determine whether the relative abundance of proteins associated with raft membranes is altered by potential homeostatic changes occurring upon loss of FMRP expression. To examine whether the composition of the brain lipid raft proteome is altered in absence of FMRP, we used a well-characterized animal model of FXS—Fmr1 knockout mice (Fmr1 KO; [55]). To this end, we isolated detergent-resistant membranes (DRMs; [49]) from the cerebrum of adult wild type (WT) and mutant Fmr1 knockout (Fmr1 KO) mice to recover sufficient material for proteome analysis. We used anti-Flotillin-1 antibodies to identify membrane fractions enriched with lipid rafts and anti-Transferrin receptor 1 (TfR1) antibodies to visualize gradient fractions containing non-raft membranes. Flotillin-1 is abundantly expressed in the brain and its selective association with raft membranes has been extensively characterized in both primary neurons and brain tissue [56] and thus represents a reliable marker of lipid raft enrichment. In contrast, TfR1 is excluded from lipid raft membranes under resting or stimulated conditions [57] in both primary cells and native tissue and thus affords a reliable indicator of non-raft membranes. We found Flotillin-1 to be consistently enriched in three buoyant gradient fractions (fractions 3 to 5; Fig 1A) in both mutant and wild type mouse brain whereas TfR1 appeared concentrated in heavy gradient fractions and virtually absent from buoyant, lipid raft enriched membranes (Fig 1B). Together, these observations indicate that DRMs purified from both Fmr1 KO and WT mouse cerebrum are overall enriched in lipid raft-associated proteins and not significantly contaminated by non-raft membranes, thus providing material of adequate purity for interrogation of the lipid raft proteome.

**Fig 2:**
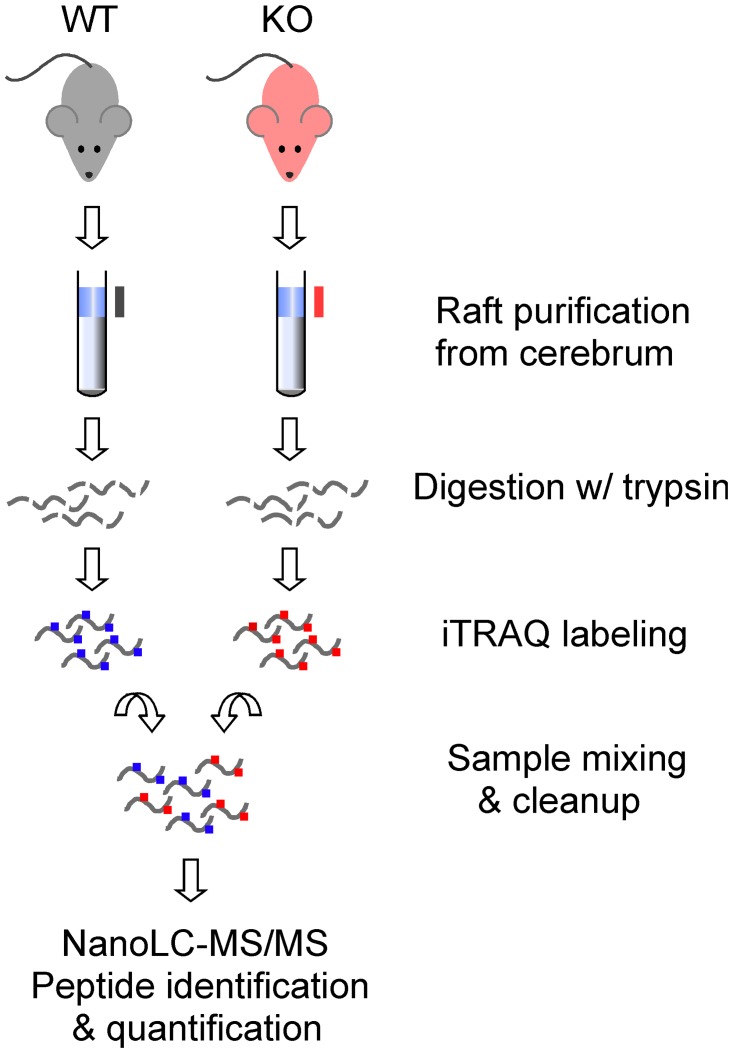
Workflow of iTRAQ proteomic strategy. Illustration of principal steps for qualitative and quantitative proteomic profiling of brain DRMs.

To systematically investigate the composition of the lipid raft proteome in Fmr1 KO mice and quantitatively compare the relative abundance of raft-associated proteins to WT mice, we used isobaric tags for relative and absolute quantification (iTRAQ; [47])-based mass spectrometry (Fig 2). We chose iTRAQ because it affords concurrent identification and quantification of peptides in complex mixtures in solution and can be applied to purified sub-cellular fractions. Moreover, iTRAQ does not require gel matrix-based protein isolation that may compromise recovery of integral membrane proteins and hydrophobic polypeptides. Peptides differentially labeled with iTRAQ tags are chromatographically indistinguishable due to mass conservation but when fragmented by tandem mass-spectrometry analysis a balance group is released and unmasked reporter ions allow relative quantification of peaks in mass spectra.

To implement iTRAQ workflow (Fig 2), DRM-containing gradient fractions were pooled to obtain sufficient material while decreasing individual variability: proteolytic peptides of mutant or wild type DRMs were independently labeled with different iTRAQ tags and then combined into one sample mixture that was analyzed by MS/MS. Three independent experiments were performed with mice from different litters: obtained peptide matches above identity threshold were 575 (FDR 1.22%), 796 (FDR 1.63%) and 257 (FDR 0.00%), respectively. High-confidence peptides for either genotypes identified 201, 293 and 140 protein hits from individual experiments, in good agreement with estimated raft proteome complexity of ~ 200 proteins [58, 59]. For data analysis, we combined independently obtained 2-plex datasets and compiled a list of 133 candidate proteins present in brain lipid rafts (Table 1) that includes proteins identified in both genotypes by high confidence peptides in at least one experiment (S1 Table). Overall, the derived representation of the mouse brain lipid raft proteome is concordant with proteomic studies previously performed employing alternative strategies [39, 58, 60–62] or tissue samples including neonatal mouse brain [58] and human brain tissue [62]. To evaluate the functions associated with proteins present in the lipid raft proteome of adult mouse brain, high confidence raft proteins identified in all three experiments (85 proteins; Table 2) were used to search the Database for Annotation, Visualization and Integrated Discovery (DAVID; [50, 51]) that allows identification of over-represented Gene Ontology (GO) terms in query protein/gene sets. Bioinformatics analysis with DAVID revealed that proteins present in brain lipid rafts were mostly associated with plasma membrane as expected (Table 3) but also enriched at synapses and cell projections and significantly associated with heterotrimeric G protein complexes. Molecular functions (Table 3) over-represented in the lipid raft proteome included ion and proton transport, ATPase and GTPase activity and component of myelin sheath. Moreover, brain lipid raft proteins appeared to be significantly associated with important biological processes (Table 3) involved in neuronal function and maturation including, but not limited to, synaptic transmission and cell adhesion.

**Table 1:** Lipid raft proteome of adult mouse brain.

#	UniProtKB	Gene symbol	Gene ID	Description	*	**	^	^^	#
**1**	1433B	Ywhab	54401	14-3-3 protein beta/alpha	√				
**2**	1433E	Ywhae	22627	14-3-3 protein epsilon					√
**3**	1433G	Ywhag	22628	14-3-3 protein gamma		√			√
**4**	1433Z	Ywhaz	22631	14-3-3 protein zeta/delta	√	√			
**5**	AATM	Got2	14719	Aspartate aminotransferase, mitochondrial		√			
**6**	ACTB	Actb	11461	Actin, cytoplasmic 1	√	√			√
**7**	ACTC	Actc1	11464	Actin, alpha cardiac muscle 1					
**8**	ACTN1	Actn1	109711	Actinin-1					
**9**	ACTN2	Actn2	11472	Actinin-2					
**10**	ADT1	Slc25a4	11739	ADP/ATP translocase 1	√	√	√		
**11**	ADT2	Slc25a5	11740	ADP/ATP translocase 2	√	√			
**12**	AGK	Agk	69923	Acylglycerol kinase, mitochondrial					
**13**	AINX	Ina	226180	Alpha-internexin					
**14**	ALDOA	Aldoa	11674	Fructose-bisphosphate aldolase A		√			
**15**	ANS1B	Anks1b	77531	Ankyrin repeat and sterile alpha motif domain-containing protein 1B					
**16**	AT1A1	Atp1a1	11928	Na/K-transporting ATPase subunit alpha-1	√				
**17**	AT1A3	Atp1a3	232975	Na/K-transporting ATPase subunit alpha-3	√	√			
**18**	AT1B1	Atp1b1	11931	Sodium/potassium-transporting ATPase subunit beta-1	√	√	√		
**19**	ATPA	Atp5a1	11946	ATP synthase subunit alpha, mitochondrial	√		√		√
**20**	ATPB	Atp5b	11947	ATP synthase subunit beta, mitochondrial	√	√	√		√
**21**	BAIP2	Baiap2	108100	Brain-specific angiogenesis inhibitor 1-assoc. protein 2	√				
**22**	BASP1	Basp1	70350	Brain acid soluble protein 1	√				
**23**	BSN	Bsn	12217	Protein bassoon			√		
**24**	C1QBP	C1qbp	12261	Complement component 1 Q subcomponent-binding protein (mito)				√	
**25**	CA2D1	Cacna2d1	12293	Voltage-dependent Ca channel subunit alpha-2/delta-1	√	√	√		
**26**	CA2D2	Cacna2d2	56808	Voltage-dependent Ca channel subunit alpha-2/delta-2				√	
**27**	CA2D3	Cacna2d3	12294	Voltage-dependent Ca channel subunit alpha-2/delta-3					
**28**	CAD13	Cdh13	12554	Cadherin-13	√				
**29**	CALM	Calm1	12313	Calmodulin					
**30**	CLCB	Cltb	74325	Clathrin light chain B				√	
**31**	CLCN6	Clcn6	26372	Chloride transport protein 6	√				
**32**	CLD11	Cldn11	18417	Claudin-11					
**33**	CLH1	Cltc	67300	Clathrin heavy chain 1	√	√			
**34**	CMC1	Slc25a12	78830	Calcium-binding mitochondrial carrier protein Aralar1	√				
**35**	CN37	Cnp	12799	2',3'-cyclic-nucleotide 3'-phosphodiesterase				√	
**36**	CNTN1	Cntn1	12805	Contactin-1	√			√	
**37**	CNTN2	Cntn2	21367	Contactin-2	√	√	√		
**38**	CNTP1	Cntnap1	53321	Contactin-associated protein 1			√		
**39**	COX2	Mtco2	17709	Cytochrome c oxidase subunit 2	√	√			
**40**	COX5A	Cox5a	12858	Cytochrome c oxidase subunit 5A, mitochondrial	√				
**41**	CSPG2	Vcan	13003	Versican core protein				√	
**42**	CTNA2	Ctnna2	12386	Catenin alpha-2	√				
**43**	DLG4	Dlg4	13385	Disks large homolog 4		√			
**44**	DLGP3	Dlgap3	242667	Disks large-associated protein 3					
**45**	DPYL2	Dpysl2	12934	Dihydropyrimidinase-related protein 2	√				
**46**	DYL1	Dynll1	56455	Dynein light chain 1, cytoplasmic				√	
**47**	ENPP6	Enpp6	320981	Ectonucleotide pyrophosphatase/phosphodiesterase member 6					
**48**	ERC2	Erc2	238988	ERC protein 2					√
**49**	FLOT1	Flot1	14251	Flotillin-1	√		√		
**50**	FLOT2	Flot2	14252	Flotillin-2	√				
**51**	G3P	Gapdh	14433	Glyceraldehyde-3-phosphate dehydrogenase				√	
**52**	GBB1	Gnb1	14688	Guanine nucleotide-binding protein G(I)/G(S)/G(T) subunit beta-1	√	√			
**53**	GBG12	Gng12	14701	Guanine nucleotide-binding protein G(I)/G(S)/G(O) subunit gamma12	√				
**54**	GBG2	Gng2	14702	Guanine nucleotide-binding protein G(I)/G(S)/G(O) subunit gamma2	√				
**55**	GNAI1	Gnai1	14677	Guanine nucleotide-binding protein G(i) subunit alpha1	√	√			
**56**	GNAI2	Gnai2	14678	Guanine nucleotide-binding protein G(i) subunit alpha2		√			
**57**	GNAO	Gnao1	14681	Guanine nucleotide-binding protein G(o) subunit alpha	√	√			
**58**	GNAS1	Gnas	14683	Guanine nucleotide-binding protein G(s) subunit alpha isoforms XLas	√				
**59**	GNAZ	Gnaz	14687	Guanine nucleotide-binding protein G(z) subunit alpha	√	√			
**60**	HOME1	Homer1	26556	Homer protein homolog 1		√	√		
**61**	HPLN1	Hapln1	12950	Hyaluronan and proteoglycan link protein 1					
**62**	HPLN2	Hapln2	73940	Hyaluronan and proteoglycan link protein 2					
**63**	HS71L	Hspa1l	15482	Heat shock 70 kDa protein 1-like		√			
**64**	HSP7C	Hspa8	15481	Heat shock cognate 71 kDa protein	√	√			√
**65**	HXK1	Hk1	15275	Hexokinase-1		√			
**66**	IGS21	Igsf21	230868	Immunoglobulin superfamily member 21					
**67**	IQEC1	Iqsec1	232227	IQ motif and SEC7 domain-containing protein 1					
**68**	IQEC2	Iqsec2	245666	IQ motif and SEC7 domain-containing protein 2					
**69**	KCC2A	Camk2a	12322	Ca/calmodulin-dependent protein kinase type II α sub.		√		√	
**70**	KCC2B	Camk2b	12323	Ca/calmodulin-dependent protein kinase type II β sub.		√		√	
**71**	KCC2D	Camk2d	108058	Ca/calmodulin-dependent protein kinase type II δ sub.					
**72**	KCC2G	Camk2g	12325	Ca/calmodulin-dependent protein kinase type II γ sub.		√			
**73**	KCRB	Ckb	12709	Creatine kinase B-type	√				√
**74**	LSAMP	Lsamp	268890	Limbic system-associated membrane protein	√				
**75**	LY6H	Ly6h	23934	Lymphocyte antigen 6H	√				
**76**	MAP1A	Map1a	17754	Microtubule-associated protein 1A	√				
**77**	MBP	Mbp	17196	Myelin basic protein		√			
**78**	MDHM	Mdh2	17448	Malate dehydrogenase, mitochondrial	√				
**79**	ML12B	Myl12b	67938	Myosin regulatory light chain 12B					
**80**	MOBP	Mobp	17433	Myelin-associated oligodendrocyte basic protein		√			
**81**	MOG	Mog	17441	Myelin-oligodendrocyte glycoprotein		√	√		
**82**	MYH10	Myh10	77579	Myosin-10	√	√			
**83**	MYH9	Myh9	17886	Myosin-9					
**84**	MYL6	Myl6	17904	Myosin light polypeptide 6					
**85**	MYO5A	Myo5a	17918	Unconventional myosin-Va					
**86**	MYPR	Plp1	18823	Myelin proteolipid protein					
**87**	NCAM1	Ncam1	17967	Neural cell adhesion molecule 1	√		√		
**88**	NCAN	Ncan	13004	Neurocan core protein				√	
**89**	NDUA4	Ndufa4	17992	NADH dehydrogenase 1 alpha subcomplex sub. 4	√	√			
**90**	NEGR1	Negr1	320840	Neuronal growth regulator 1	√				
**91**	NEUM	Gap43	14432	Neuromodulin	√	√			
**92**	NFL	Nefl	18039	Neurofilament light polypeptide					
**93**	NFM	Nefm	18040	Neurofilament medium polypeptide					
**94**	NTRI	Ntm	235106	Neurotrimin	√	√			
**95**	ODO2	Dlst	78920	Dihydrolipoyllysine-residue succinyltransferase component of 2-oxoglutarate dehydrogenase complex (mito)		√			
**96**	ODP2	Dlat	235339	Dihydrolipoyllysine-residue acetyltransferase component of pyruvate dehydrogenase complex (mito)		√			√
**97**	OMGP	Omg	18377	Oligodendrocyte-myelin glycoprotein			√		
**98**	PCLO	Pclo	26875	Protein piccolo					
**99**	PRIO	Prnp	19122	Major prion protein		√			
**100**	RAC1	Rac1	19353	Ras-related C3 botulinum toxin substrate 1	√				
**101**	SCAI	Scai	320271	Protein SCAI					
**102**	SEPT7	Sept7	235072	Septin-7		√		√	
**103**	SHAN3	Shank3	58234	SH3 and multiple ankyrin repeat domains protein 3		√			
**104**	SNP25	Snap25	20614	Synaptosomal-associated protein 25	√	√			
**105**	SPTA2	Sptan1	20740	Spectrin alpha chain, brain	√	√			
**106**	SPTB2	Sptbn1	20742	Spectrin beta chain, brain 1		√			
**107**	STX1B	Stx1b	56216	Syntaxin-1B	√	√			√
**108**	SYGP1	Syngap1	240057	Ras GTPase-activating protein SynGAP		√			
**109**	SYPH	Syp	20977	Synaptophysin		√			
**110**	SYT1	Syt1	20979	Synaptotagmin-1	√	√			
**111**	SYT2	Syt2	20980	Synaptotagmin-2					
**112**	TBA1A	Tuba1a	22142	Tubulin alpha-1A chain		√			√
**113**	TBA1B	Tuba1b	22143	Tubulin alpha-1B chain	√				√
**114**	TBA3	Tuba3a	22144	Tubulin alpha-3 chain					
**115**	TBA4A	Tuba4a	22145	Tubulin alpha-4A chain					
**116**	TBB2A	Tubb2a	22151	Tubulin beta-2A chain		√			√
**117**	TBB3	Tubb3	22152	Tubulin beta-3 chain	√				
**118**	TBB4A	Tubb4a	22153	Tubulin beta-4A chain	√				
**119**	TBB4B	Tubb4b	227613	Tubulin beta-4B chain	√				
**120**	TBB5	Tubb5	22154	Tubulin beta-5 chain	√				
**121**	THY1	Thy1	21838	Thy-1 membrane glycoprotein	√	√	√		
**122**	TPM3	Tpm3	59069	Tropomyosin alpha-3 chain					
**123**	VA0D1	Atp6v0d1	11972	V-type proton ATPase subunit d 1	√	√			
**124**	VAMP2	Vamp2	22318	Vesicle-associated membrane protein 2	√	√			
**125**	VATA	Atp6v1a	11964	V-type proton ATPase catalytic subunit A		√	√		
**126**	VATB2	Atp6v1b2	11966	V-type proton ATPase subunit B, brain isoform	√		√		
**127**	VATC1	Atp6v1c1	66335	V-type proton ATPase subunit C 1		√	√		
**128**	VATD	Atp6v1d	73834	V-type proton ATPase subunit D			√		
**129**	VATE1	Atp6v1e1	11973	V-type proton ATPase subunit E 1		√	√		
**130**	VATF	Atp6v1f	66144	V-type proton ATPase subunit F					
**131**	VATG2	Atp6v1g2	66237	V-type proton ATPase subunit G 2					
**132**	VDAC1	Vdac1	22333	Voltage-dependent anion-selective channel protein 1	√	√	√		√
**133**	VPP1	Atp6v0a1	11975	V-type proton ATPase 116 kDa subunit a isoform 1	√	√	√		

(√), Identified in corresponding proteomic studies: * [58], ** [61], ^ [62], ^^ [60], # [39]. List of all proteins (133) identified in DRMs from adult WT and Fmr1 KO mouse brain. List includes proteins identified in three, two or one experiment. Entry descriptors: UniProtKB, UniProt Knowledgebase database (UniProtKB/Swiss-Prot) *Mus musculus* protein entry name; gene symbol, *Mus musculus*; Entrez Gene ID, National Center for Biotechnology Information (NCBI) Entrez *Mus musculus* gene ID. Columns annotated with symbols (*, **, ^, ^^, #) correspond to proteomic studies listed in appended references that reported identification of the corresponding protein in lipid rafts.

**Table 2:** Quantitative profiling of lipid raft proteome in WT and Fmr1 KO mouse brain.

#	UniProtKB	Gene symbol	Entrez ID	Ave KO/WT	Sdev	***P*-value**		Description
1	1433B	Ywhab	54401	0.99	0.375	0.909	ns	14-3-3 protein beta/alpha
2	AATM	Got2	14719	1.07	0.537	0.865	ns	Aspartate aminotransferase, mitochondrial
3	ACTB	Actb	11461	0.97	0.110	0.560	ns	Actin, cytoplasmic 1
4	ACTC	Actc1	11464	0.99	0.124	0.758	ns	Actin, alpha cardiac muscle 1
5	ADT1	Slc25a4	11739	0.90	0.240	0.485	ns	ADP/ATP translocase 1
6	ADT2	Slc25a5	11740	0.95	0.254	0.712	ns	ADP/ATP translocase 2
7	ANS1B	Anks1b	77531	1.05	0.099	0.588	ns	Ankyrin repeat and sterile alpha motif domain-containing protein 1B
8	AT1A3	Atp1a3	232975	1.08	0.021	0.180	ns	Sodium/potassium-transporting ATPase subunit alpha-3
9	AT1B1	Atp1b1	11931	1.22	0.601	0.591	ns	Sodium/potassium-transporting ATPase subunit beta-1
10	ATPA	Atp5a1	11946	0.92	0.121	0.304	ns	ATP synthase subunit alpha, mitochondrial
11	ATPB	Atp5b	11947	0.91	0.164	0.366	ns	ATP synthase subunit beta, mitochondrial
12	BAIP2	Baiap2	108100	1.29	0.335	0.237	ns	Brain-specific angiogenesis inhibitor 1-associated protein 2
13	BASP1	Basp1	70350	1.13	0.070	0.101	ns	Brain acid soluble protein 1
14	BSN	Bsn	12217	1.13	0.112	0.204	ns	Protein bassoon
15	C1QBP	C1qbp	12261	0.99	0.217	0.886	ns	Complement component 1 Q subcomponent-binding protein, (mito)
16	CA2D1	Cacna2d1	12293	0.99	0.111	0.736	ns	Voltage-dependent calcium channel subunit alpha-2/delta-1
17	CA2D3	Cacna2d3	12294	0.79	0.137	0.065	ns	Voltage-dependent calcium channel subunit alpha-2/delta-3
18	CAD13	Cdh13	12554	0.93	0.130	0.376	ns	Cadherin-13
19	CALM	Calm1	12313	0.85	0.169	0.186	ns	Calmodulin
20	CLH1	Cltc	67300	0.92	0.205	0.479	ns	Clathrin heavy chain 1
21	CN37	Cnp	12799	1.32	0.244	0.101	ns	2',3'-cyclic-nucleotide 3'-phosphodiesterase
22	CNTN1	Cntn1	12805	0.89	0.125	0.203	ns	Contactin-1
23	CNTN2	Cntn2	21367	0.88	0.222	0.363	ns	Contactin-2
24	COX2	Mtco2	17709	1.04	0.158	0.776	ns	Cytochrome c oxidase subunit 2
25	COX5A	Cox5a	12858	1.02	0.491	0.974	ns	Cytochrome c oxidase subunit 5A, mitochondrial
**26**	**CSPG2**	**Vcan**	**13003**	**0.82**	**0.079**	**0.031**	*	**Versican core protein**
27	DLG4	Dlg4	13385	0.94	0.236	0.631	ns	Disks large homolog 4
28	ENPP6	Enpp6	320981	1.22	0.340	0.366	ns	Ectonucleotide pyrophosphatase/phosphodiesterase 6
**29**	**ERC2**	**Erc2**	**238988**	**1.17**	**0.066**	**0.042**	*	**ERC protein 2**
30	FLOT1	Flot1	14251	0.80	0.146	0.084	ns	Flotillin-1
31	FLOT2	Flot2	14252	1.10	0.101	0.280	ns	Flotillin-2
32	GBB1	Gnb1	14688	0.76	0.264	0.186	ns	Guanine nucleotide-binding protein G(I)/G(S)/G(T) subunit beta-1
33	GBG12	Gng12	14701	1.29	0.860	0.608	ns	Guanine nucleotide-binding protein G(I)/G(S)/G(O) subunit gamma-12
34	GBG2	Gng2	14702	1.28	0.226	0.121	ns	Guanine nucleotide-binding protein G(I)/G(S)/G(O) subunit gamma-2
35	GNAO	Gnao1	14681	1.19	0.229	0.259	ns	Guanine nucleotide-binding protein G(o) subunit alpha
36	GNAS1	Gnas	14683	1.14	0.361	0.581	ns	Guanine nucleotide-binding protein G(s) subunit alpha isoforms XLas
37	GNAZ	Gnaz	14687	1.12	0.251	0.516	ns	Guanine nucleotide-binding protein G(z) subunit alpha
38	HOME1	Homer1	26556	1.31	0.280	0.148	ns	Homer protein homolog 1
39	HSP7C	Hspa8	15481	1.01	0.064	>0.999	ns	Heat shock cognate 71 kDa protein
40	IGS21	Igsf21	230868	1.01	0.083	0.918	ns	Immunoglobulin superfamily member 21
41	KCC2A	Camk2a	12322	1.27	0.299	0.215	ns	Calcium/calmodulin-dependent protein kinase type II subunit alpha
42	KCC2B	Camk2b	12323	1.24	0.365	0.343	ns	Calcium/calmodulin-dependent protein kinase type II subunit beta
43	KCC2G	Camk2g	12325	1.21	0.331	0.362	ns	Calcium/calmodulin-dependent protein kinase type II subunit gamma
44	KCRB	Ckb	12709	0.85	0.124	0.108	ns	Creatine kinase B-type
45	LSAMP	Lsamp	268890	0.94	0.142	0.443	ns	Limbic system-associated membrane protein
**46**	**LY6H**	**Ly6h**	**23934**	**0.76**	**0.020**	**0.003**	**	**Lymphocyte antigen 6H**
47	MAP1A	Map1a	17754	1.06	0.097	0.499	ns	Microtubule-associated protein 1A
48	MBP	Mbp	17196	1.35	0.318	0.144	ns	Myelin basic protein
49	MDHM	Mdh2	17448	0.85	0.327	0.444	ns	Malate dehydrogenase, mitochondrial
50	MOBP	Mobp	17433	1.47	0.345	0.089	ns	Myelin-associated oligodendrocyte basic protein
51	MOG	Mog	17441	1.31	0.185	0.060	ns	Myelin-oligodendrocyte glycoprotein
52	MYH10	Myh10	77579	1.02	0.245	0.966	ns	Myosin-10
53	MYH9	Myh9	17886	1.14	0.668	0.760	ns	Myosin-9
54	MYO5A	Myo5a	17918	1.05	0.136	0.720	ns	Unconventional myosin-Va
**55**	**MYPR**	**Plp1**	**18823**	**1.55**	**0.176**	**0.008**	**	**Myelin proteolipid protein**
56	NCAM1	Ncam1	17967	1.00	0.128	0.880	ns	Neural cell adhesion molecule 1
57	NCAN	Ncan	13004	1.02	0.427	0.990	ns	Neurocan core protein
58	NEGR1	Negr1	320840	1.01	0.209	0.980	ns	Neuronal growth regulator 1
59	NTRI	Ntm	235106	0.97	0.101	0.566	ns	Neurotrimin
60	ODO2	Dlst	78920	1.15	0.267	0.448	ns	Dihydrolipoyllysine succinyltransferase comp. of 2-oxoglutarate dehydrogenase complex
61	ODP2	Dlat	235339	1.03	0.150	0.894	ns	Dihydrolipoyllysine acetyltransferase comp. of pyruvate dehydrogenase complex
62	OMGP	Omg	18377	1.12	0.044	0.076	ns	Oligodendrocyte-myelin glycoprotein
63	PCLO	Pclo	26875	1.08	0.015	0.140	ns	Protein piccolo
64	PRIO	Prnp	19122	0.84	0.142	0.126	ns	Major prion protein
65	RAC1	Rac1	19353	1.28	0.381	0.293	ns	Ras-related C3 botulinum toxin substrate 1
66	SCAI	Scai	320271	1.46	0.971	0.471	ns	Protein SCAI
67	7-Sep	Sept7	235072	1.45	0.272	0.052	ns	Septin-7
68	SHAN3	Shank3	58234	1.01	0.284	0.970	ns	SH3 and multiple ankyrin repeat domains protein 3
69	SNP25	Snap25	20614	1.10	0.551	0.800	ns	Synaptosomal-associated protein 25
70	SPTA2	Sptan1	20740	0.97	0.157	0.681	ns	Spectrin alpha chain, brain
71	SPTB2	Sptbn1	20742	0.97	0.099	0.530	ns	Spectrin beta chain, brain 1
72	STX1B	Stx1b	56216	1.05	0.445	0.895	ns	Syntaxin-1B
73	SYGP1	Syngap1	240057	1.09	0.106	0.344	ns	Ras GTPase-activating protein SynGAP
74	SYT1	Syt1	20979	0.90	0.236	0.456	ns	Synaptotagmin-1
75	TBA1B	Tuba1b	22143	1.21	0.155	0.113	ns	Tubulin alpha-1B chain
**76**	**TBB2A**	**Tubb2a**	**22151**	**1.29**	**0.155**	**0.045**	*	**Tubulin beta-2A chain**
**77**	**TBB5**	**Tubb5**	**22154**	**1.27**	**0.125**	**0.034**	*	**Tubulin beta-5 chain**
**78**	**THY1**	**Thy1**	**21838**	**0.66**	**0.132**	**0.014**	*	**Thy-1 membrane glycoprotein**
79	VA0D1	Atp6v0d1	11972	1.27	0.255	0.163	ns	V-type proton ATPase subunit d 1
80	VAMP2	Vamp2	22318	1.12	0.715	0.810	ns	Vesicle-associated membrane protein 2
81	VATA	Atp6v1a	11964	1.05	0.137	0.697	ns	V-type proton ATPase catalytic subunit A
82	VATB2	Atp6v1b2	11966	1.04	0.090	0.662	ns	V-type proton ATPase subunit B, brain isoform
83	VATE1	Atp6v1e1	11973	1.17	0.232	0.314	ns	V-type proton ATPase subunit E 1
84	VDAC1	Vdac1	22333	1.00	0.367	0.954	ns	Voltage-dependent anion-selective channel protein 1
85	VPP1	Atp6v0a1	11975	1.19	0.156	0.144	ns	V-type proton ATPase 116 kDa subunit a isoform 1

List of proteins (85) identified and quantified in three independent experiments in WT and Fmr1 KO brain DRMs. Numbers highlighted in bold correspond to proteins with significantly different abundance in FMR1 KO rafts. Indicated are mean ratio of Fmr1 KO *vs*. WT (KO/WT), standard deviation (Sdev) and *p*-value (t-test) in relation to internal reference (HSP7C, mean ± sdev, 1.01 ± 0.064), with *p* <0.05 considered significant. Entry descriptors: UniProtKB, UniProt Knowledgebase database (UniProtKB/Swiss-Prot) *Mus musculus* protein entry name; gene symbol, *Mus musculus*; Entrez Gene ID, National Center for Biotechnology Information (NCBI) Entrez *Mus musculus* gene ID.

**Table 3:** Gene ontology annotation of brain lipid raft proteome.

GOTERM _CC	Term	Count	%	P value	Fold enrich.	Bonferroni	Benjamini
GO:0005886	Plasma membrane	48	56.47	5.34E-11	2.45	1.09E-08	1.09E-08
GO:0031225	Anchored to membrane	14	16.47	9.19E-10	10.07	1.88E-07	9.37E-08
GO:0045202	Synapse	13	15.29	1.18E-06	6.07	2.41E-04	3.44E-05
GO:0005834	Heterotrimeric G-protein complex	6	7.06	3.17E-06	25.52	6.46E-04	8.08E-05
GO:0042995	Cell projection	16	18.82	4.84E-06	4.14	9.87E-04	9.87E-05
**GOTERM _MF**	**Term**						
GO:0015077	Monovalent inorganic cation transmembrane transporter activity	10	11.76	4.54E-10	22.46	7.77E-08	7.77E-08
GO:0005516	Calmodulin binding	10	11.76	5.16E-09	17.14	8.82E-07	4.41E-07
GO:0015078	Hydrogen ion transmembrane transporter activity	9	10.59	7.21E-09	21.45	1.23E-06	4.11E-07
GO:0042625	ATPase activity, coupled to transmembrane movement of ions	8	9.41	5.06E-08	22.66	8.66E-06	1.73E-06
GO:0003924	GTPase activity	9	10.59	2.41E-07	13.74	4.12E-05	5.89E-06
GO:0016887	ATPase activity	11	12.94	1.31E-06	7.65	2.23E-04	1.86E-05
GO:0019911	Structural constituent of myelin sheath	3	3.5	7.49E-05	195.41	0.0127	8.53E-04
**GOTERM _BP**	**Term**						
GO:0006754	ATP biosynthetic process	9	10.59	1.21E-08	20.15	9.99E-06	9.99E-06
GO:0019226	Transmission of nerve impulse	12	14.12	3.04E-08	9.75	2.50E-05	8.33E-06
GO:0015985	Energy coupled proton transport, down electrochemical gradient	7	8.24	3.97E-08	34.74	3.27E-05	4.67E-06
GO:0007268	Synaptic transmission	10	11.76	4.38E-07	10.32	3.60E-04	1.80E-05
GO:0006119	Oxidative phosphorylation	7	8.24	5.12E-07	22.95	4.21E-04	2.00E-05
GO:0006812	Cation transport	14	16.47	3.07E-06	4.99	0.002	1.05E-04
GO:0007155	Cell adhesion	14	16.47	7.80E-06	4.58	0.006	2.29E-04

Gene ontology (GO) terms annotation conducted with DAVID classification system (v. 6.7) using a list of proteins (85) identified and quantified in three independent experiments in WT and Fmr1 KO brain DRMs and *Mus musculus* genome reference background. Selected GO terms for Cellular Components (GOTERM_CC), Molecular Function (GOTERM_MF) and Biological Process (GOTERM_BP) over-represented in genes included in brain raft proteome. *Term*, GO term description; *Count*, number of genes annotated with a given term; *%*, percentage of genes annotated with a given term; *P value*, significance of gene-term enrichment; *Fold enrichment*, geometric mean of all the enrichment *P* values of associated terms; *Bonferroni* and *Benjamini-Hochberg*, *P*-value correction. Redundant or broad GO terms associated with many genes are not listed.

### Identification of proteins differentially enriched in brain lipid rafts of Fmr1 KO mice

To estimate with a degree of stringency differential raft association of individual protein hits in WT v*s*. Fmr1 KO mice, we restricted quantitative analysis to those proteins for which high confidence peptides were identified in each of three independent datasets (Table 2). We calculated for each protein a Fmr1 KO/WT ratio, with a ratio of 1 indicating no change and ratios >1 or <1 indicating increased or decreased raft association in Fmr1 KO brain, respectively and compared average ratios to an internal reference protein with ratio ~1 in all experiments. Although the majority of analyzed proteins appeared similarly represented in DRMs from Fmr1 KO *vs*. WT mice, seven proteins (~8.2% of quantified proteome) displayed statistically significant (mean ± SEM, *N* = 3, *p* <0.05) differential abundance (Table 2). Among identified candidates, three showed decreased abundance in Fmr1 KO rafts (Lymphocyte antigen 6H, human *locus* LY6H, chromosome 8q24.3; Thy-1 membrane glycoprotein, human *locus* THY1, 11q23.3; CSPG2/Versican core protein, human *locus* VCAN, 5q14.2) whereas four were increased (ERC protein 2, human *locus* ERC2, chromosome 3p14.3; Myelin proteolipid protein/Plp1, human *locus* PLP1, Xq22; Tubulin beta-2A chain, human *locus* TUBB2A, 6p25.2; Tubulin beta 5 chain, human *locus* TUBB, 6p21.33). Interestingly, although the lipid raft proteome appeared enriched in proteins encoded by RNA transcripts that are potential FMRP targets [8]- 41 proteins of 133 present in the raft proteome (~31%; Table 4)—only one, Plp1 [8], showed differential abundance in DRMs (~1.2% of 85 quantified proteins).

**Table 4:** Lipid raft associated proteins in WT and Fmr1 KO brain encoded by FMRP targets.

#	Gene symbol
1	Actb
2	Aldoa
3	Atp1a1
4	Atp1a3
5	Atp1b1
6	Atp5a1
7	Atp5b
8	Atp6v0a1
9	Atp6v0d1
10	Atp6v1b2
11	Bsn
12	Calm1
13	Camk2a
14	Camk2b
15	Ckb
16	Cltc
17	Cnp
18	Dlg4
19	Dlgap3
20	Dpysl2
21	Gnao1
22	Gnas
23	Gnaz
24	Gnb1
25	Hk1
26	Iqsec2
27	Mbp
28	Myh10
29	Myo5a
30	Ncam1
31	Ncan
32	Pclo
33	Plp1
34	Sept4
35	Shank3
36	Snap25
37	Syngap1
38	Syt1
39	Tubb3
40	Vamp2
41	Ywhag

Identified raft proteins that are encoded by RNA transcripts regulated by FMRP, based on a genome-wide HITS-CLIP study in mouse brain [8].

**Fig 3:**
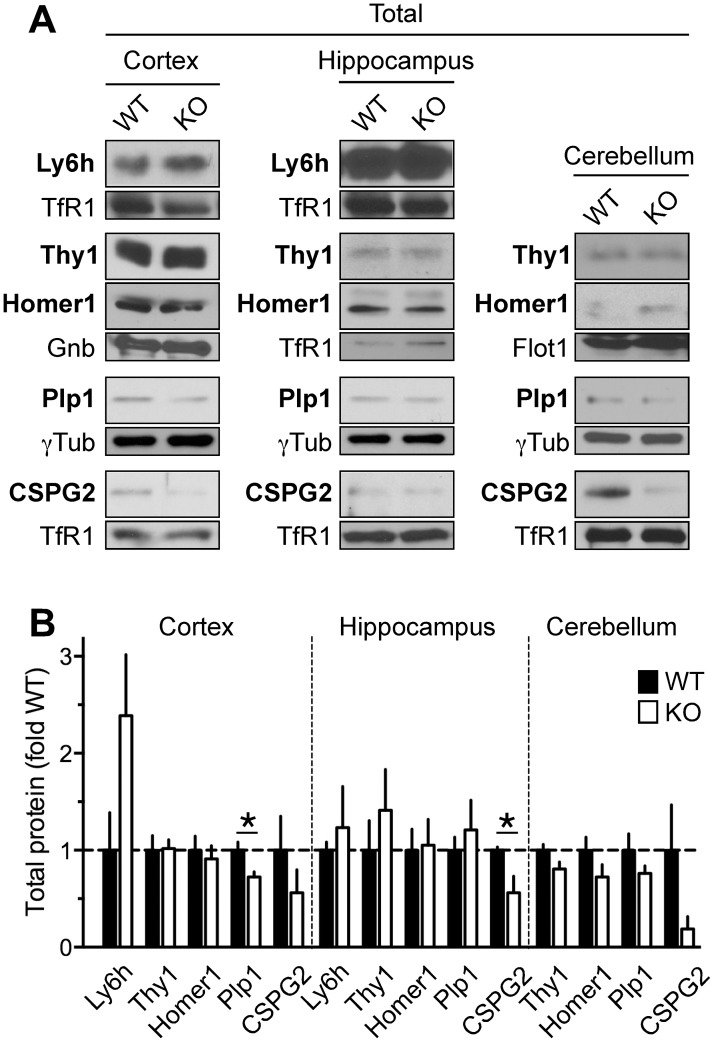
Brain expression of candidate protein hits in Fmr1 KO and WT mice. A) Representative immunoblots of extracts from cortices, hippocampi and cerebella of Fmr1 KO and WT mice probed with antibodies anti- Homer-1b/c (~ 45 kDa), -Ly6h (~15 kDa), -Plp1 (~ 32 kDa), Thy-1 (~ 25–37 kDa) and CSPG2 (~ 60 kDa). Protein loading was estimated by probing with either anti-TfR1, -γTubulin, -Flotillin or—Gnb2 depending on gel separation. B) Quantification of relative abundance of indicated proteins from experiments like those in (A) measured as ratio of loading control and normalized to WT. Means ± SEM, *N* = 3 for all groups except Homer-1b/c (cortex), Plp1 and CSPG2 (hippocampus) with N = 4. Unpaired t-test, *p <0.05.

**Fig 4:**
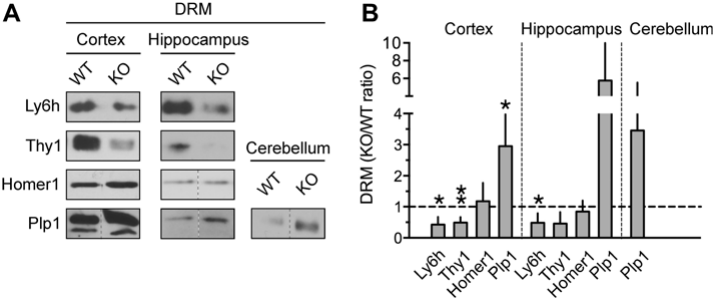
Abundance of candidate protein hits in DRMs of Fmr1 KO and WT mice. A) Representative immunoblots of combined DRM enriched fractions from individual cortices and cerebella or hippocampi pooled from two animals per group probed with antibodies anti- Homer-1b/c, -Ly6h, -Plp1 or-Thy-1. B) Quantification of relative abundance in DRMs of indicated proteins from experiments like those in (A) normalized to Flotillin content. Calculated KO/WT ratios of candidate proteins were compared to control Flotillin (KO/WT ratio, mean ± SEM 0.997 ± 0.033, *N* = 3). Means ± SEM, *N* = 3 for all groups; unpaired t-test, *p <0.05.

To independently validate changes in association with lipid rafts detected by iTRAQ, we examined the abundance of selected candidate proteins in extracts (Fig 3) or DRMs (Fig 4) from cortex, hippocampus and cerebellum of individual Fmr1 KO and WT mice by Western blot. We focused on candidate proteins (Ly6h, Thy1, Plp1, CSPG2) that displayed more robust changes and for which specific antibodies are available. As control, we also examined the abundance of Homer-1b/c, a scaffold protein that interacts with Gp1 mGluRs [63] and the expression of which was shown to be unaffected in Fmr1 KO mouse brain [64, 65]. We found that expression of Ly6h, Thy-1 and Homer-1b/c did not significantly differ between genotypes in all brain regions examined (Fig 3) but noted a trend to increased abundance of Ly6h in the Fmr1 KO cortex that did not achieve statistical significance (*p* = 0.1338). In contrast, Plp1 and CSPG2 showed significantly reduced abundance in Fmr1 KO mice in cortex and hippocampus, respectively (Fig 3). Analysis of CSPG2, which is encoded by different splice isoforms, was limited to the V3 form that could be readily detected in our conditions *vs*. other isoforms of very high molecular mass (>400 kDa). Since Plp1 is a potential FMRP target and alterations in protein levels in Fmr1 KO mice might arise from abnormal translation of mRNAs that bind FMRP, we also examined whether an independent FMRP target—CamKIIα[66, 67]—displayed similarly altered expression and observed no significant difference in Fmr1 KO compared to WT (S1 Fig), in agreement with others [67].

Consistent with quantitative mass spectrometry, we found that Ly6h and Thy-1 abundance in DRMs was significantly reduced in the cortex of Fmr1 KO mice *vs*. WT (Fig 4) and observed a similar decrease in the hippocampus although in the case of Thy-1 it did not reach statistical significance (KO/WT, *p* = 0.0664). We further confirmed the presence of Homer-1b/c in lipid raft-enriched membranes with similar abundance in Fmr1 KO and WT mouse cortex and hippocampus (Fig 4). Whereas Ly6h is not expressed in the cerebellum [68], we were unable to estimate Thy-1 and Homer-1b/c abundance in DRMs from cerebellum likely due to low expression in adult animals (Fig 3; [69]). In agreement with proteomic findings, Plp1 showed significantly increased abundance in DRMs from Fmr1 KO cortices (Fig 4) and appeared similarly increased in hippocampus and cerebellum in which though it did not reach statistical significance. CSPG2 expression in DRMs was not examined because the molecular complexity of native Versican (the form detected by mass spectrometry) precluded accurate analysis of high molecular mass isoforms in our experimental conditions. Collectively, these observations suggest that changes in lipid raft composition are not likely to be simply dictated by protein expression levels but may instead also arise from broader cellular dysfunctions in FMRP lacking cells.

**Fig 5:**
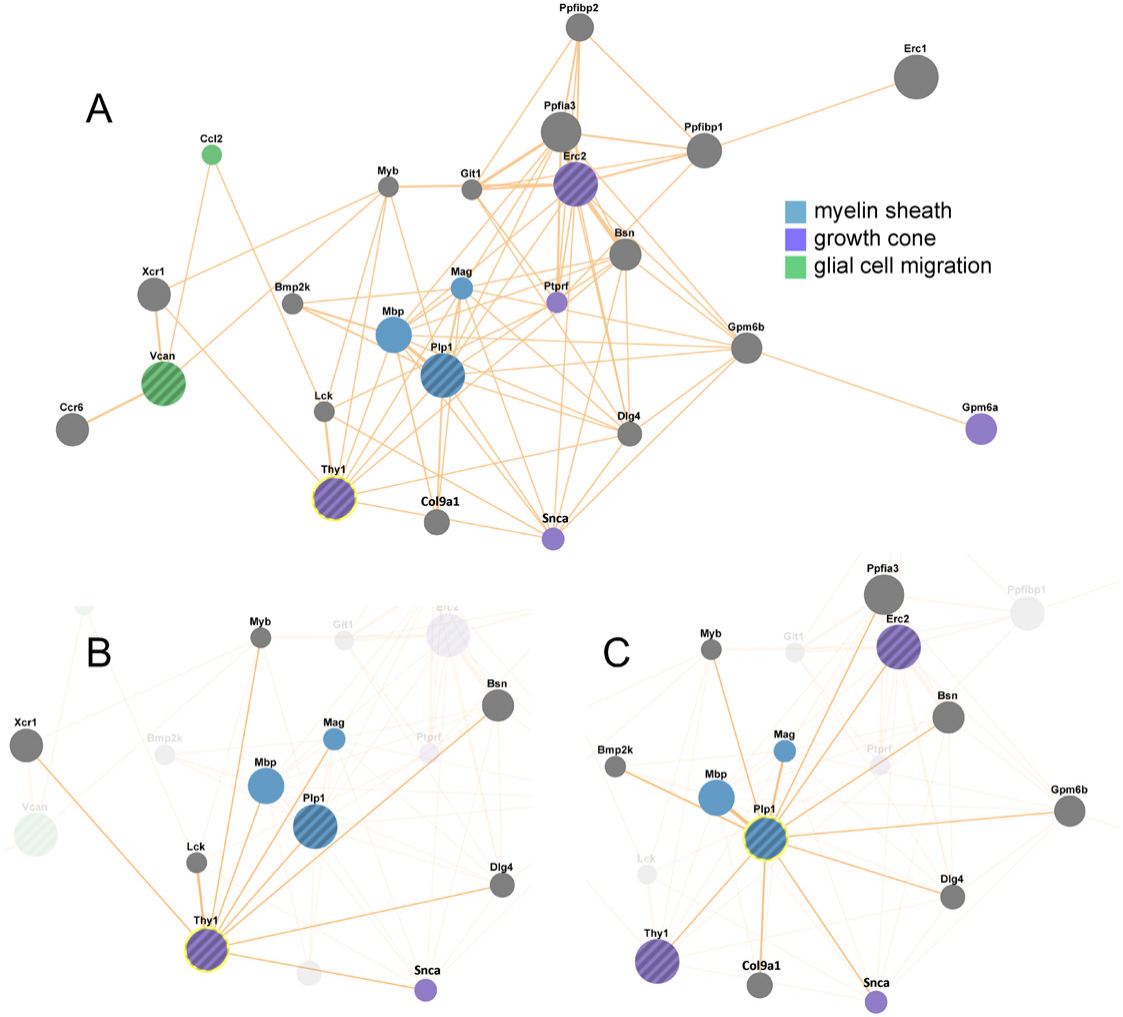
Network analysis of selected proteins differentially enriched in brain lipid rafts of Fmr1 KO mice. Network analysis constructed with GeneMANIA using four (Erc2, Plp1, Thy1, Vcan) of seven identified genes encoding proteins differentially expressed in brain lipid rafts of Fmr1 KO mice. Connections (cellular component based) are predicted based on annotated databases and indicated by links whereby thickness conveys degree of confidence of the relationship. A) Combined network view including four query genes with selected functions highlighted in color: myelin sheath (FDR 1.13e-2), growth cone (FDR 5.89e-4), glial cell migration (FDR 7.41e-2). Additional functions include axon part (FDR 4.17e-4), calcium ion transport ((FDR 8.98e-4), regulation of synaptic transmission (FDR 4.39e-3), positive regulation of lymphocyte activation (FDR 2.2.8e-2). B, C) Network views highlighting neighbours of Thy1 (B) or Plp1 (C).

Biological networks provide a valuable framework to integrate diverse information on protein properties arising from genome-wide proteomic studies and are particularly effective in enabling association of individual proteins/genes to complex functions. To obtain information on molecular networks and pathways that might be affected by abnormalities in lipid rafts, we performed network analysis with GeneMANIA [52] using as query four identified candidate genes: Erc2, Plp1, Thy1 and Vcan. Tubb2a and Tubb5 were omitted from combined analysis because of their broad functionality that could skew network weight, whereas we were unable to integrate Ly6h in pathways analysis due to paucity of molecular information available for this gene. Interestingly, network analysis highlighted connections between identified genes and predicted further functional connectivity with genes encoding components of myelin sheath, axon growth cones (and terminals) and axonal processes (Fig 5). Altogether, our findings could inform prioritization of studies aimed to identify salient molecular and cellular dysfunctions arising from Fmr1 silencing and potentially contribute to uncovering of shared cellular pathologies between FXS and other disorders in the autism spectrum.

## Discussion

Here, we used an unbiased, genome-wide proteomic strategy to define the brain lipid raft proteome of Fmr1 knockout mice—an animal model of Fragile X syndrome—and test the hypothesis that raft association of subsets of proteins might be affected by homeostatic changes occurring in absence of FMRP. We found that whereas overall proteome representation (~93%) is conserved, a discrete subset of proteins displays significantly altered abundance in DRMs of Fmr1 KO mice. Gene ontology and network analysis of proteins displaying abnormal abundance in absence of FMRP suggest convergence onto common cellular components and biological processes, such as axonal processes and myelination, emphasizing novel aspects of the complex biology of Fragile X syndrome and potentially other disorders in the autism spectrum.

The raft proteome shows an estimated maximum complexity of approximately 200 proteins based on studies carried out with different technologies and biological samples including HeLa cells [59], neonatal mouse brain [58] and human brain tissue [62]. Results from our study applying stringent criteria for protein assignment, identify 133 proteins in lipid-raft enriched membrane fractions of adult mouse brain, consistent with expected raft proteome complexity, and illustrate robust proteome coverage by iTRAQ despite incomplete peptide overlap among experiments that precluded quantitative analysis of all identified proteins. Observed variance in relative protein abundance reported by iTRAQ was modest (highest ratio difference ~1.5-fold, lowest ~0.6-fold) but of magnitude consistent with biologically relevant differences. Although iTRAQ offers a high degree of precision, it does not provide a measure of ‘absolute’ changes in protein abundance [47]: moreover, ratio compression can also arise from the fact that DRMs are a complex heterogeneous mixture of membranes extracted from different brain regions and cell types with potentially different properties.

We found that approximately 48% of the raft proteome, for which we could apply quantitation, is encoded by FMRP mRNA targets. Surprisingly, although absence of FMRP can lead to enhanced mRNA translation we found that only one identified FMRP target shows significantly increased abundance in DRMs of Fmr1 KO mice. Several mRNA targets of FMRP [8] encode proteins involved in lipid homeostasis, including lipid synthesis (*e*.*g*. PIGQ, FASN, SCD2, AGPAT3), metabolism (*e*.*g*. LPIN2, LPPR4, CPT1A, SMPD3), and cholesterol homeostasis (*e*.*g*. SREBF2, SCAP) and transport (*e*.*g*. LPR1, SORL1, ABCA3, LRP8). For example, PIGQ is involved in early steps of GPI anchor biosynthesis [70] and deficiencies in this pathway are linked to Mabry syndrome [71] marked by severe developmental delays and intellectual disability, autosomal recessive intellectual disability [72] and intractable epilepsy [73]. Notably, two GPI-linked proteins—Thy-1 and Ly6h —display altered abundance in Fmr1 KO rafts, suggesting the possibility that GPI biosynthesis may be compromised in absence of FMRP. Involvement of potential dysfunctions in lipid homeostasis in FXS is further suggested by the findings that HMG-CoA reductase inhibitors that reduce cholesterol synthesis partly correct pathological dysfunctions in Fmr1 KO mice [74] and decrease Gp1 mGluR signaling to ERK1/2 in Fmr1 KO null neurons [57], lending indirect support for a potential role of the cholesterol biosynthetic pathway in FXS manifestations, at least in animal models.

An interesting observation arising from quantitative proteomics is that whereas a subset of proteins showed increased abundance in DRMs of Fmr1 KO mice, a group of proteins including Thy-1 and Ly6h displayed decreased raft association. Thy-1 is a developmentally regulated IgG-like protein enriched in neurons [75]. Although its precise functions and mechanisms of action remain unclear, Thy-1 was shown to inhibit neurite outgrowth and stabilize neuronal processes [76] *via* both cis- and trans-interactions between neuronal Thy-1 and α_V_β_3_ integrin in astrocytes, a mechanism contributing to Thy-1 impact on neurite outgrowth [77]. Importantly, lipid raft integrity and intact GPI anchor are required for Thy-1 actions. Thy1 knockout mice show impaired LTP in dentate gyrus [78] and behavioral abnormalities such as impaired social transmission of food preference [79] that are consistent with a critical function for this *locus* in neurodevelopment and neurodevelopmental disorders [80]. Ly6h, a GPI-anchored protein, is a member of the Ly6 complex—a protein family involved in immune regulation—encoded by ≥ 20 genes many of which cluster in human chromosome 8q24.3, a hot spot of deletion/duplication events linked to ASD and attention deficit hyperactivity disorder [81]. Ly6h expression in neurons is developmentally regulated and spatially restricted to specific brain regions (*e*.*g*. frontal cortex, hippocampus [68]). Although its function in the brain is unknown, the closely related Ly6/Neurotoxin 1 (Lynx1) was shown to modulate nAChRs functional properties and stabilize mature cortical networks [82]. Interestingly, peptides corresponding to Lynx1 were identified in DRMs by our study but not included in proteome data analysis on quality grounds. Outside the CNS, both Ly6h and Thy-1 are expressed in immune related cells including human cortical thymocytes and lymphoblastoid cell lines, suggesting the possibility that they could represent novel biomarkers for FXS and potentially other ASDs. In addition to GPI-linked proteins, another protein showing decreases raft association in Fmr1 KO mice is the versican core protein CSPG2/Vcan, a proteoglycan and a major component of extracellular matrix, with functions in cells adhesion and presynaptic maturation [83] among others.

Several proteins displayed increased abundance in brain rafts from Fmr1 KO mice, most prominently Plp1, an abundant component of CNS myelin, which plays a critical function in myelin formation and maintenance, oligodendrocyte development and axonal survival [84]. Mutations in PLP1 are linked to Pelizaeus-Merzbacher disease and spastic paraplegia (SPG2), conditions associated with intellectual disability and ASD [85]. Proteomic profiling also identified other protein components of the myelin sheath including CNP, MBP, MOBP and MOG all of which displayed a trend (not statistically significant) to increased abundance in Fmr1 KO rafts (Table 2). Consistent with this, network analysis highlights myelin sheath as a potential convergent pathway for identified protein with abnormal abundance in Fmr1 KO rafts. Myelination abnormalities are detected in FXS patients [86] and recent evidence indicates that FMRP is expressed in oligodendrocytes [87] and Fmr1 KO mice display delayed myelination during development [88]. Together, these observations suggest that dysfunctions of myelination at the cellular and/or circuitry level may play an important and unappreciated role in pathological manifestations of FXS.

An additional subset of proteins also appeared to be modestly increased in rafts from mutant Fmr1 mice including two tubulin beta chain isoforms, encoded by Tubb5 and Tubb2a respectively, and ERC2. Tubulin has been shown to associate with lipid rafts and disruption of the microtubule cytoskeleton can lead to remodeling of raft membrane composition [53]. ERC2 is enriched at the presynaptic active zone and implicated in organization of the cytomatrix at synaptic terminals [89]: interestingly, the closely related ERC1 is associated with ASD [90].

The precise mechanisms underlying altered raft association in Fmr1 KO mice are at present unclear and they could involve changes in overall protein abundance or abnormalities in protein trafficking. Moreover, since DRMs are a heterogeneous mixture of membranes they do not allow to distinguish whether observed changes occur in defined cell populations and whether they are cell-autonomous or dependent on cell adhesion or trophic conditions in intact circuitry. Use of orthogonal strategies will be needed in the future to address these questions and further determine whether abnormalities in raft association reflect homeostatic adaptation in the adult brain (used in this study) and/or are present at early stages of brain development.

## Supporting Information

S1 Fig Brain expression of CaMKIIα/β in Fmr1 KO and WT mice. A) Representative immunoblots of cortical extracts from WT and Fmr1 KO mice probed with anti-CaMKIIα/β and pan-β Tubulin antibodies. B) Quantification of CaMKIIα/β relative abundance from experiments like those in (A) measured as ratio of band densities for CaMKIIα/β *vs*. tubulin and normalized to WT: means ± SEM, *N* = 3, unpaired t-test, *p* = 0.157 and *p* = 0.203 for CaMKIIαand CaMKIIβ respectively.
(TIF)

S1 Table List of peptides identified in WT and Fmr1 KO mouse brain. Number of peptide matches and corresponding protein identified in three (A) two (B) or one (C) experiment. Columns E1-3 indicate number of peptide matches obtained for each protein hit: numbers in brackets indicate number of peptide used for ratio when calculated.
(XLS)
