# Supplementary figures and images for: Quantitative Profiling of Brain Lipid Raft Proteome in a Mouse Model of Fragile X Syndrome
Source: PLoS One. 2015 Apr 7;10(4):e0121464. doi: 10.1371/journal.pone.0121464 (PMC4388542; doi:10.1371/journal.pone.0121464)

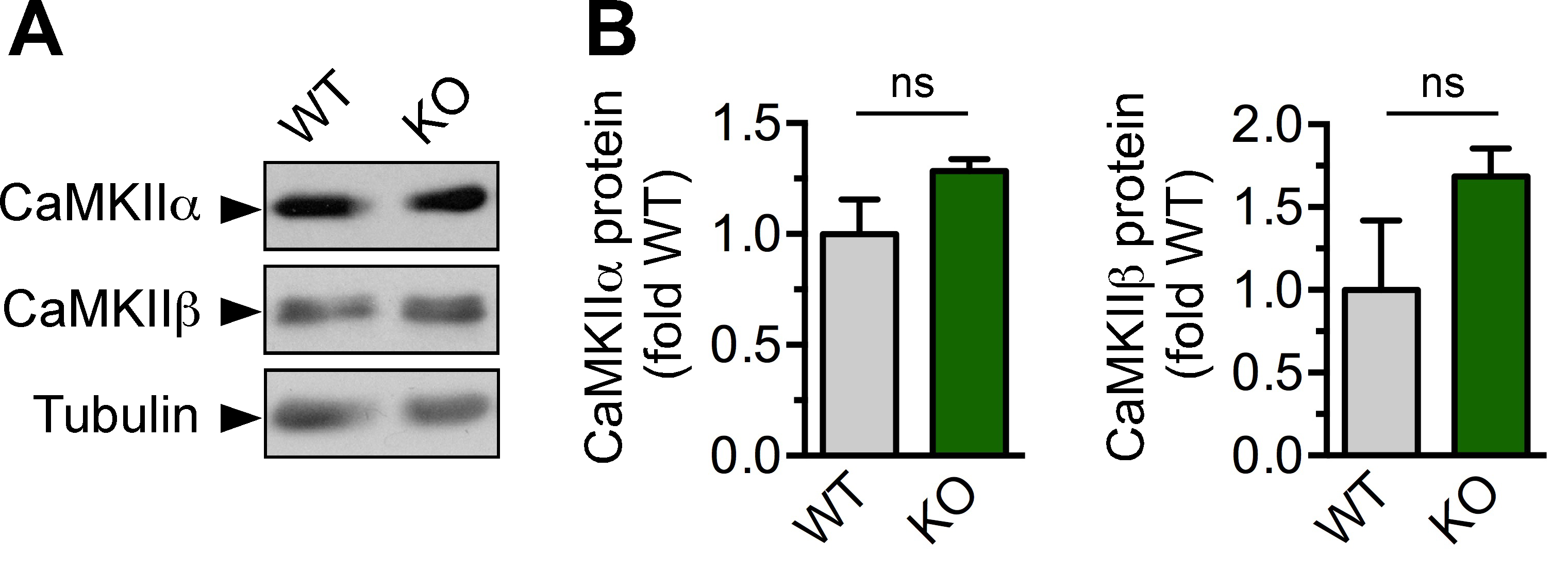

Supplement: S1 Fig — A) Representative immunoblots of cortical extracts from WT and Fmr1 KO mice probed with anti-CaMKIIα/β and pan-β Tubulin antibodies. B) Quantification of CaMKIIα/β relative abundance from experiments like those in (A) measured as ratio of band densities for CaMKIIα/β vs. tubulin and normalized to WT: means ± SEM, N = 3, unpaired t-test, p = 0.157 and p = 0.203 for CaMKIIαand CaMKIIβ respectively. (TIF) [file pone.0121464.s001.tif]
